# PTGDR -441 C/T Polymorphism in a Mexican Mestizo Population with Inflammatory Myopathies: A Pilot Study

**DOI:** 10.3390/cimb48060561

**Published:** 2026-05-28

**Authors:** Mónica Vázquez-Del Mercado, Beatriz Teresita Martín-Márquez, Erika Aurora Martínez-García, Marcelo Heron Petri

**Affiliations:** 1 Departamento de Biología Molecular y Genómica, Instituto de Investigación en Reumatología y del Sistema Músculo Esquelético, Centro Universitario de Ciencias de la Salud, Universidad de Guadalajara, Guadalajara C.P. 44340, Jalisco, Mexico; dravme@hotmail.com (M.V.-D.M.); beatriz.martin@academicos.udg.mx (B.T.M.-M.); erikaaurora21@hotmail.com (E.A.M.-G.); 2 División de Medicina Interna, Servicio de Reumatología SNP 004086 de la SECIHTI, Nuevo Hospital Civil Dr. Juan I. Menchaca, Guadalajara C.P. 44340, Jalisco, Mexico; 3 Cuerpo Académico UDG-CA-703 “Inmunología y Reumatología”, Centro Universitario de Ciencias de la Salud, Universidad de Guadalajara, Guadalajara C.P. 44340, Jalisco, Mexico; 4 Departamento de Fisiología, Centro Universitario de Ciencias de la Salud, Universidad de Guadalajara, Sierra Mojada No 950, Colonia Independencia, Guadalajara C.P. 44340, Jalisco, Mexico; 5 Department of Medicine Solna, Karolinska Institute, 17176 Stockholm, Sweden; 6 School of Medical Sciences, Örebro University, 70182 Örebro, Sweden

**Keywords:** inflammatory myopathies, prostaglandin D2 receptor, polymorphism

## Abstract

The prostaglandin D2 receptor (PTGDR) -441 C/T polymorphism has been previously associated with inflammatory diseases such as asthma. The goal of this study was to explore the association of this polymorphism with idiopathic inflammatory myopathies (IIM) and their clinical features. Seventy-two healthy subjects (HS) and forty-eight patients with IIM from Guadalajara and Mexico were recruited over three years. Clinical features and enzymes used for diagnostics and follow-up, such as creatine phosphokinase (CPK), lactate dehydrogenase (LDH), aspartate transaminase (AST), and alanine aminotransferase (ALT), were collected at the recruitment point and from the chart at the diagnostic point. PCR-ARMS was used to define genotypes. The Chi-square test was used to compare genotype and allele frequencies and clinical features between IIM patients and healthy subjects. A one-way ANOVA on ranks was performed to compare enzymatic levels. The allele distribution was not in Hardy–Weinberg equilibrium in the controls. There was no difference in age or gender distribution between the groups. The polymorphic (T) allele was more common in the IIM group than in the HS group. At the same time, the wild-type (CC) genotype presented more clinical features, such as heliotrope rash, fever, dyspnea, and weight loss, than the TT genotype. No significant differences were found regarding the enzyme levels. To further understand the role of this polymorphism in IIM, a bigger sample size is required along with mechanistical studies. Nevertheless, the polymorphic allele of the PTGDR -441 C/T polymorphism suggests susceptibility to IIM, whereas the wild-type CC genotype is associated with more clinical features.

## 1. Introduction

Idiopathic inflammatory myopathies (IIMs), such as polymyositis (PM) and dermatomyositis (DM), are characterized by muscle weakness and infiltration of both CD4^+^ and CD8^+^ T lymphocytes into the inflamed muscle, leading to muscle damage and autoantibody production [1]. In IIM, the muscle presents important cellular infiltration, mainly of T cells. The muscle damaged by the immune system releases enzymes such as creatine phosphokinase (CPK), lactate dehydrogenase (LDH), aspartate transaminase (AST), and alanine aminotransferase (ALT) into the bloodstream. These enzymes are commonly used for clinical diagnosis and follow-up [2,3]. IIM patients often have a specific clinical feature when they have a particular myositis-specific antibody (MSA). According to the evolution of the classification criteria since their first description by Bohan and Peter in 1975 [2], the clinical phenotypes have been revised by the European League Against Rheumatism and the American College of Rheumatology (EULAR/ACR) 2017 [3] and the European Neuromuscular Centre (ENMC) [4,5] as follows: DM, PM, inclusion body myositis (IBM) and immune-mediated necrotizing myopathy (IMNM), overlap myositis (OV), cancer-associated myositis (CAM) and anti-synthetase syndrome (ASS) taking into account the adult population.

Genetic background may play a role in the pathogenesis of IIM, as the prevalence of autoimmune diseases in first-degree relatives is around 22% [4]. Given the limited genetic studies on IIM, there is a clear need for further research to uncover genetic factors. Since it is a rare disease and commonly misdiagnosed it is crucial to explore the genetic predisposition to such disease. The Mexican mestizo population used in this work was originally established to describe antibodies that present specific clinical features and antibody profiles [6]. During this study, DNA was also collected and was previously shown to be associated with Alpha Actinin 3 (ACTN3) [5] and Interferon-*γ* [7] polymorphisms. This emphasizes the need to explore the impact of genetic susceptibility on this disease.

The prostaglandin D2 receptor (PTGDR) gene is located on chromosome 14 at q22.1 and contains four introns and five exons, leading to a protein of approximately 44 kilodaltons [8]. It is a membrane G-protein-coupled receptor that binds primarily to prostaglandin D2 (an omega-6 derivative), a mast cell-derived lipid mediator with pro-inflammatory properties, which has been associated with asthma [9,10]. The receptor is also expressed in basophils, eosinophils, Th2 cells, dendritic cells, and vascular endothelium cells, suggesting a crucial role in immune response [11]. Studies performed in PTGDR^−/−^ mice revealed that it mediates T-cell chemotaxis and decreases inflammation, mainly associated with Th2 cytokines, lymphocyte accumulation in the lungs, and immunoglobulin levels, upon ovalbumin challenge [10]. Studies of genetic variation across populations aim to clarify how PTGR influences asthma risk, address the controversy over inconsistent findings, and highlight the importance of genetic diversity in research.

Three variants in the PTGDR promoter region have been shown to affect the C/EBPβ promoter, a transcription factor associated with asthma in the white American population [12]. Among these, the PTGDR -441 C/T (rs803010) polymorphism showed a mechanistic effect on the PTGDR receptor, whereas the T variant led to reduced PTGDR expression, modulating inflammation under corticoid treatment [12]. For this reason, this work focused on this variant. Such polymorphism was also associated with asthma in the north Indian population [13], whereas no association was found in the Australian population [14], Puerto Ricans or African Americans [15]. In the Mexican mestizo population, it was also not associated with asthma [15]. Nevertheless, this population was the only one that did not follow the Hardy–Weinberg equilibrium (HWE) among the groups studied. To our knowledge, there is no information about this or any other PTGDR polymorphism in autoimmune or rheumatic diseases.

We hypothesized that the PTGDR -441 C/T polymorphism influences disease development or activity in IIM, based on its association with other inflammatory diseases, such as asthma, across populations. To test this, genetics, clinical features, and enzymatic levels were examined in a cohort of IIM patients from two major Mexican cities over three years.

## 2. Materials and Methods

### 2.1. Patients

Subjects for this study were recruited from Guadalajara and Mexico City. The protocol was approved by the Institutional Review Board (IRB) committee of Hospital Civil de Guadalajara “Dr. Juan I. Menchaca”, under the register 969/10, and all subjects signed an informed written consent. Forty-eight patients with IIM (12 PM and 36 DM), who fulfilled the Bohan and Peter criteria [2], were recruited from March 2009 until May 2012. Their clinical features were recorded at the time of recruitment. As a control group, we included 72 healthy subjects (HS) matched by gender and age. Since it is a rare disease and this was an exploratory study, no power analysis was performed; data from all patients were collected continuously for three years.

### 2.2. Laboratory Studies

Blood was drawn by peripheral venipuncture for general studies including blood enzymes such as CPK, LDH, AST, and ALT, which were recorded from the time of diagnosis (retrieved from the chart as starting levels) and at the time of recruitment (as current).

### 2.3. Genotyping

Blood was collected in EDTA, and DNA was extracted using the Miller modified technique [16]. The DNA was stored at −20 °C until PCR analysis. The genotypes were analyzed by PCR-ARMS as described elsewhere [13] using the outer primers 5-TCTGTAGCTCTGTCACCACGGGCAGATCAC-3 and 5-CTGACGCGCTGCGTTCT CAGTAGA-GACAGA-3 as a control and the inner primers 5-AAGCA GCAGCCACCTGAGAGGAGGGAAG-3 for the C allele and 5-GCCACCCCCAGTTCA AACACCAGCACAAT-3 for the T allele. The PCR product was verified on 4% agarose gels stained with GelRed^®^ (Biotium, Fremont, CA, USA) and used to confirm the band pattern. The outer primers as a positive control yielded a 434 bp fragment; if the C allele was present, a 295 bp fragment was obtained, and if the T allele was present, a 195 bp fragment was obtained. If the patient was heterozygous (CT), three fragments of 434, 295 and 195 bp were present. As a negative control a PCR mix was placed without DNA. To ensure the results, 10% of the genotypes were randomly selected and tested in a second round to confirm those that showed a 100% match. No sequencing was done.

### 2.4. Statistical Analysis

The HWE was computed in the controls. Fisher’s exact test was used to compare genotype and allele frequencies, as well as clinical features, between IIM patients and HS participants. A one-way ANOVA on ranks (Kruskal–Wallis’s test) was used to compare the levels of muscle enzymes among the genotypes. A *p*-value of <0.05 was considered significant. All analyses were performed with SigmaPlot (version 12.0, Palo Alto, CA, USA).

## 3. Results

### 3.1. Demographic Distribution and Time with the Disease

Thirty-one males and forty-one females were recruited as HS, all of them from Guadalajara due to logistical constraints. The patients with IIM were 10 males and 26 females with DM (seven from Guadalajara) and seven males and five females with PM (two from Guadalajara). There was no difference in age distribution among the patients with DM and PM and the HS, as shown in Table 1.

### 3.2. Prevalence of the PTGDR -441 C/T Polymorphism

The genotype distribution is expressed in Table 2. The distribution in the HS was CC = 26 (36%), CT = 22 (29%) and TT = 24 (35%). Even with this small sample size these genotypes were not in HWE (*p* < 0.05). In the IIM group the genotype was as follows: CC = 9 (19%), CT = 13 (27%) and TT = 26 (54%). The Chi-square test showed that the TT genotype was significantly more common in the disease group. On the other hand, comparing the type of IIM (DM vs. HS or PM vs. HS) we found significant differences between groups. In an analysis of the alleles, in the HS group, C = 74 (51%) and T = 70 (49%) were significantly different by Fisher’s exact test when compared with the IIM group, with C = 31 (32%) and T = 65 (68%), with *p* < 0.05 (OR = 2.21 IC 95% 1.29–3.78).

### 3.3. PTGDR -441 C/T and Enzymatic Levels of CPK, LDH, AST and ALT in Blood

CPK, LDH, AST, and ALT in U/L were grouped by the time of data collection, at the diagnostic time (starting) retrieved from the chart or at the time of recruitment for the study (current). Highlighting these significant differences over time aims to reassure the consistency of enzyme-level changes, even though differences were found between genotypes (Table 3).

### 3.4. PTGDR -441 C/T and Clinical Features of IIM

The clinical features commonly found in patients with IIM, such as Shaw sign, heliotrope, Gottrön, dyspnea, fever and weight loss, were analyzed across genotypes as a whole group. Most of those features were more common in the wild-type genotype compared with the polymorphic genotype (Table 4). The shawl sign was more common in the TC genotype than in the TT genotype. By type of myopathy, PM patients could not be analyzed, as they did not show significance due to the low number of patients. On the other hand, among DM patients, significance persisted: Gottrön, dyspnea, fever and weight loss were more common in the CC genotype than the TT genotype (data not shown).

## 4. Discussion

The current work highlights three distinct results regarding PTGDR -441 C/T polymorphism. First, the Mexican mestizo population appears to not follow the HWE. Second, the polymorphic allele was associated with IIM development, along with the TT genotype, whereas the wild-type genotype was associated with clinical features. Third, females seem to have a longer time to diagnosis than males, which would lead to a less aggressive disease.

The current work showed that the control groups did not follow the HWE. This is the second report on the PTGDR -441 C/T polymorphism in the Mexican mestizo population. The first one focused on Mexican populations with asthma and showed that this polymorphism was not in HWE [13], suggesting that the Mexican population is mestizo and does not follow Mendelian inheritance. Given that Spanish conquistadors introduced the European genotype into the native population over the years and that North Americans also influenced the current Mexican genotype it is reasonable to conclude that these findings may only indicate that the polymorphic allele is widely distributed in this population. Interestingly, this same control population was used in two previous studies on different polymorphisms, and they followed the HWE, highlighting the importance of these insights for researchers and medical professionals interested in population genetics and epidemiology [5,7]. We acknowledge that the control group deviated from HWE at the PTGDR -441 CT locus. While technical errors such as primer design flaws or cross-contamination were ruled out through internal validation and negative controls, this deviation represents a significant limitation. It is important to recognize that these results are not definitive and must be validated through further studies. It likely reflects a combination of localized genetic sub-structure (population stratification) at this specific locus and the relatively small control sample size (*n* = 72). Consequently, these genetic associations must be interpreted with caution and serve strictly as exploratory, pilot data that require validation in larger, multi-centric populations. Lipid mediators and their receptors are poorly studied in rheumatic diseases; therefore, there is a need for studies exploring this signaling pathway. Many polymorphisms of PTGDR have been described, but the current work was focused on only one. It is important to take these results with reservations, and a larger sample size would be crucial to confirming this observation. Previous studies focusing on PTGDR -441 C/T were mainly asthma-specific and showed an association only in the North Indian population. On the other hand, the Australian population [14], Puerto Ricans and African Americans [15] failed to show any association of asthma with this polymorphism.

Although the polymorphic allele is well represented and distributed in the Mexican mestizo population, the current work, despite its limited sample size, suggests that the polymorphic allele and genotype may play a role in the development of IIM. When the analysis was stratified by disease subtype (PM and DM). Due to the rarity of IIM, our stratified analysis by clinical subtype (PM vs. DM) lacked statistical power, rendering separate comparisons for the PM cohort (*n* = 12) inconclusive. Furthermore, the overall association between the total IIM group and HS was borderline significant (*p* = 0.047). Highlighting this study’s exploratory status, this borderline significance suggests caution in interpretation, as it may fluctuate or vanish with a larger cohort. This underscores the preliminary nature of our study and the critical need for expanded replication cohorts. A major limitation of this study is the limited number of recruited patients, particularly regarding the specific genetic variants examined. In genetic studies of rare diseases, borderline significance in small sample sizes frequently serves as an exploratory signal that requires cautious interpretation, rather than definitive proof of association [16,17]. To mitigate this, we included five different centers in Mexico City and Guadalajara and extended the recruitment period over three years. Even with these challenges, our findings were sufficient to show that the polymorphic T allele was 2.2 times more common in the disease group than the HS. It is essential to promote additional studies in other populations. Increasing the number of subjects with diverse genetic backgrounds will be crucial to better understanding the role of the PTGDR -441 C/T polymorphism in the pathophysiology of IIM.

Another interesting result of the current work was that the wild-type genotype was associated with clinical features. One possible explanation is that the TT genotype has been reported to be associated with receptor expression by altering the affinity of transcriptional factors for the promoter region in the nucleus [12]. As the aim of this work was to describe the impact of polymorphism in clinical settings and to increase knowledge of genetic predisposition in patients with IIM, no mechanistic experiments were conducted, which is a limitation of the current study. On the other hand, mechanistic experiments have been done in cell cultures, and the polymorphic allele decreased the expression of the PTGD receptor on the cell surface, thereby reducing the number of available receptors and decreasing inflammation [18]. This concept has been demonstrated in animal models of asthma, in which mice lacking the PTGD receptor exhibit reduced inflammation and cellular infiltration [10].

Our findings reveal an intriguing segregation pattern: the T allele/TT genotype is associated with IIM, suggesting susceptibility, whereas the wild-type CC genotype is associated with greater clinical severity. This dual-action paradox suggests that distinct genetic and immunological mechanisms govern disease occurrence and clinical progression. Functionally, the TT genotype has been reported to down-regulate cell-surface PTGDR expression [18]. We hypothesize that while low receptor expression might alter initial immune cell homing or signaling—contributing to a loss of tolerance and promoting disease onset—it paradoxically acts as an inflammatory ‘attenuator’ once the disease is established. This phenomenon has been previously characterized by other core inflammatory pathways of systemic autoimmune diseases, where variants associated with lower expression levels limit the tissue’s capacity to sustain aggressive clinical damage [19]. However, these associations may be influenced by population-specific factors or sample size limitations, which should be considered when interpreting the results and their applicability to broader patient groups. Due to the lack of function analysis in this work, we can only speculate that PTDGR expression could be linked to genotype. The clinical features, such as dyspnea, fever and weight loss, also presented differences associated with different genotypes, suggesting that the allele previously associated with higher expression of the receptor may also impact clinical features in patients with IIM. With these results, confounders such as treatments and other factors cannot be excluded from this observation, and mechanistic experiments would be important to better understanding the impact of the PTGDR -441 C/T polymorphism in IIM.

No significant differences were found in the genotypes and enzymatic levels at the diagnostic point. Unfortunately, the variability in immunosuppressive regimens, as well as differences in corticosteroid doses and treatment durations, made it difficult to normalize or adjust the data to assess the precise impact of the PTGDR -441 C/T polymorphism. As in previous analyses, the limited number of patients further reduced the statistical power. On the other hand, such a devastating disease needs to be studied even with limited numbers, and these results need to be confirmed in other populations to confirm the role of this polymorphism in IIM. Although no previous work can be used for comparison, these results agree with a previous study that explored different promoter polymorphisms, including -441, and corticoid treatment in vivo [20]. The same study also explored three other polymorphisms in the promoter region of PTGDR, showing that different haplotypes lead to changes in PTGDR expression. Because most patients were in remission and receiving corticosteroids at the time of recruitment, reduced muscle enzyme levels were expected. It is also important to note that PM and DM patients displayed different disease durations, and that female patients had a longer disease evolution than males. These factors further complicate efforts to determine the influence of immunosuppressive or corticosteroid treatment on clinical features or enzyme levels in relation to genetic variation.

Another interesting result is that women presented a longer disease duration and were more prevalent in this population. Given the limited number of patients, the results of subanalyses need to be taken with caution. Such observations may be due to the patient recruitment process. Although it was consecutive recruitment from the outpatient clinic during working hours and this disease is more common in women, a cultural effect could also affect this finding. Women usually seek medical attention during the day, whereas men do so more often at night. Another limitation is that the controls were recruited in Guadalajara, and some of the patients were collected from Mexico City to have enough patients to analyze. This could also represent a selection bias due to the recruitment process. Specifically, this introduces the potential for geographic population stratification bias. In the Mexican mestizo population, ancestral genetic proportions (European vs. Amerindian) vary geographically; western regions like Guadalajara often show different admixture ratios to central regions like Mexico City [21,22]. Therefore, noting these regional differences is essential, as they may bias the genetic association results, rather than reflecting true susceptibility to IIM.

Since the association with the PTGDR was previously linked to asthma, drug therapies with antagonists have been explored [23]. However, the PTGDR -441 C/T polymorphism exhibits remarkable genetic heterogeneity across distinct populations and pathologies. In asthma, a positive association was reported in North Indian cohorts [13]. In contrast, studies in other ethnically diverse populations, including Hispanic, Puerto Rican, and Mexican cohorts, failed to establish a significant link [15,24]. This discrepancy underscores the context-dependent pathological role of PTGDR. While asthma is an airway-localized, Th2-mediated disease in which PGD2 signaling mainly regulates eosinophil mechanics, IIM is a systemic autoimmune condition characterized by distinct cellular subsets and a prominent interferon signature. Interestingly, because our model suggests that high receptor expression in the wild-type CC genotype exacerbates systemic clinical features, selective PTGDR antagonists may have novel therapeutic potential for IIM. Specifically, patients with the CC genotype who exhibit severe clinical manifestations might benefit most from targeted PTGDR blockade to downregulate muscle and systemic inflammation. This report suggests that patients with IIM carrying the wild-type genotype, previously associated with high PTGDR expression, also show more clinical features, which could inform future diagnostic or therapeutic strategies despite current limitations. Unfortunately, this work does not support prognosis, as no differences were observed in patients’ enzymatic levels among those in remission and, as mentioned before, the number of patients was limited despite three years and two cities in which the patients were recruited.

In conclusion, even with a limited number of patients and no mechanistic experiments done, the current work suggests that the -441 C/T polymorphism of the PTGD receptor, the polymorphic genotype, could be associated with susceptibility to developing IIM. At the same time, the wild-type genotype was associated with some clinical features in patients. Acknowledging these limitations emphasizes the need for larger mechanistic studies to confirm and expand upon these findings, which is crucial for advancing our understanding of IIM genetics.

## Figures and Tables

**Table 1:** Geographic distribution with recruitment site, sex and age of IIM patients and HS, and time with disease for IIM patients.

	From Guadalajara	Males	Evolution Time (Months) Mean ± SEM	Females	Evolution Time (Months) Mean ± SEM	Age Mean ± SEM
DM	7	10	3.7 ± 0.3	26	17.2 ± 1.3	42 ± 6
PM	2	7	2.54 ± 0.5	5	23.2 ± 9.5	47 ± 4
HS	72	31		41		36 ± 5

DM, dermatomyositis; PM, polymyositis; SEM, standard error of the mean. Time from diagnosis (starting) and time of study recruitment (current) were registered, and time with disease in months was calculated and grouped by sex and type of IIM. Female patients presented a trend of longer evolution time compared to males, but such results were not significant.

**Table 2:** PTGDR -441 C/T genotype and allele distribution.

Genotypes	CC N (%)	CT N (%)	TT N (%)	*p* Value
HS	26 (36)	22 (29)	24 (35)	
DM/PM	9 (19)	13 (27)	26 (54)	0.047
DM	8 (22)	10 (28)	18 (50)	NS
PM	1 (8)	3 (25)	8 (67)	NS
**Alleles**		**C**	**T**	
HS		74 (51)	70 (49)	
DM/PM		31 (32)	65 (68)	0.003

The table represents on the top, the genotype distribution of the healthy subjects (HS) and IIM patients, DM and PM. The chi square test was used to compare the genotype of healthy against IIM and or DM/PM as individual groups. The bottom part of the table represents the allele distribution of the HS compared with IIM patients. The fisher exact test was used to compare alleles. A *p* < 0.05 was considered significant. DM: Dermatomyositis, PM: Polymyositis, not significant (NS).

**Table 3:** Enzymatic levels in IIM patients grouped by genotype and time point.

Genotypes	CC	CT	TT	*p* Value
	Median (IQR)	Median (IQR)	Median (IQR)	
**Starting**				
CPK	1250 (124.8–2774.5)	1897 (50.7–8308.5)	895 (39.9–3577)	0.799
LDH	738 (456–1037)	769 (229.5–1587.5)	460.5 (2378–891.5)	0.262
AST	152.5 (56.4–298.2)	143 (50–237.5)	125 (54.8–242.4)	0.993
ALT	120.5 (50.8–389.7)	180 (85.5–486)	98 (60–211)	0.261
**Current**				
CPK	367.5 (184.5–2095.25)	117.5 (65.7–229.5)	160.5 (107.5–396–7)	0.089
LDH	353 (244–674.5)	223 (160–440)	275 (189–415)	0.379
AST	28 (24–69.1)	23 (15–49)	29.5 (21.5–34)	0.711
ALT	33 (25.5–100.7)	28 (12–74)	25.5 (20–39.5)	0.368

The table depicts the enzymatic levels of CPK, LDH, AST and ALT in U/L at the diagnostic time (starting) and at the time of the recruitment to the study (current). Non-parametric data shown as mean and Interquartile Range (IQR). One-way ANOVA on ranks (Kruskal–Wallis H test).

**Table 4:** Clinical features of patients with IIM grouped by genotype.

	CC	CT	TT
Total	N (%) 9 (100%)	N (%) 13 (100%)	N (%) 26 (100%)
Shawl sign	5 (56)	9 (69) ^&^	13 (50) ^&^
Heliotrope	6 (67) ^¤^	7 (54)	14 (54) ^¤^
Gottron	5 (56)	8 (62)	14 (54)
Dyspnea	1 (11) ^¤^	3 (23)	6 (23) ^¤^
Fever	5 (56) ^¤^	3 (23)	5 (19) ^¤^
Weight loss	7 (78) *^¤^	4 (31) *	13 (50) ^¤^

The table represents the different clinical features present in patients with IIM grouped by genotype. The patients that represent positive features from the total are expressed on the table and a fisher exact test was applied to compare the genotypes. ^¤^ = *p* < 0.05 in TT versus CC, ^&^ = *p* < 0.05 in TT versus TC and * = *p* < 0.05 in TC versus CC.

## Data Availability

The original contributions presented in this study are included in the article. Data availability is limited and further inquiries can be directed to the corresponding author.

## Abbreviations

The following abbreviations are used in this manuscript:

ACR	American College of Rheumatology
ALT	Alanine aminotransferase
ASS	Anti-synthetase syndrome
AST	Aspartate transaminase
CAM	Cancer-associated myositis
CPK	Creatine phosphokinase
DM	Dermatomyositis
EULAR	European League Against Rheumatism
ENMC	European Neuromuscular Centre
IBM	Inclusion body myositis
IIM	Idiopathic inflammatory myopathies
IMNM	Immune-mediated necrotizing myopathy
IRB	Institutional Review Board
HWE	Hardy-Weinberg equilibrium
LDH	Lactate dehydrogenase
MSA	Myositis-specific antibody
HS	Healthy subject
OV	Overlap myositis
PM	Polymyositis
PTGDR	Prostaglandin D2 receptor
